# Supplementary material for: Immunophenotypic correlates of sustained MRD negativity in patients with multiple myeloma
Source: Nat Commun. 2023 Sep 2;14:5335. doi: 10.1038/s41467-023-40966-8 (PMC10475030; doi:10.1038/s41467-023-40966-8)
Supplement: Supplementary file 1 — Supplementary Information [file 41467_2023_40966_MOESM1_ESM.pdf]

Supplementary Information

Immunophenotypic correlates of sustained MRD negativity in patients with multiple myeloma

Target	Clone	Manufacturer	Catalog #
CD11c	BU15	Biolegend	337202
IgD	IA6-2	Biolegend	348202
CD19	REA675	Miltenyi	130-122-301
CD45RA	REA562	Miltenyi	130-122-292
CD4	REA623	Miltenyi	130-122-283
CD8	REA734	Miltenyi	130-122-281
CD16	REA423	Miltenyi	130-108-027
CD127	A019D5	Fluidigm	3149011B
CD1c	REA694	Miltenyi	130-122-298
CD123	REA918	Miltenyi	130-122-297
CD27	REA499	Miltenyi	130-122-295
CD33	WM53	Fluidigm	3158001B
CD14	REA599	Miltenyi	130-122-290
CD56	REA196	Miltenyi	130-108-016
CD25	REA570	Miltenyi	130-122-302
CCR7	G043H7	Fluidigm	3167009A
CD3	REA613	Miltenyi	130-122-282
CD38	REA671	Miltenyi	130-122-288
HLA-DR	REA805	Miltenyi	130-122-299

Supplementary Table 1. CyTOF antibody targets, clone identifiers, manufacturer, and catalog numbers.

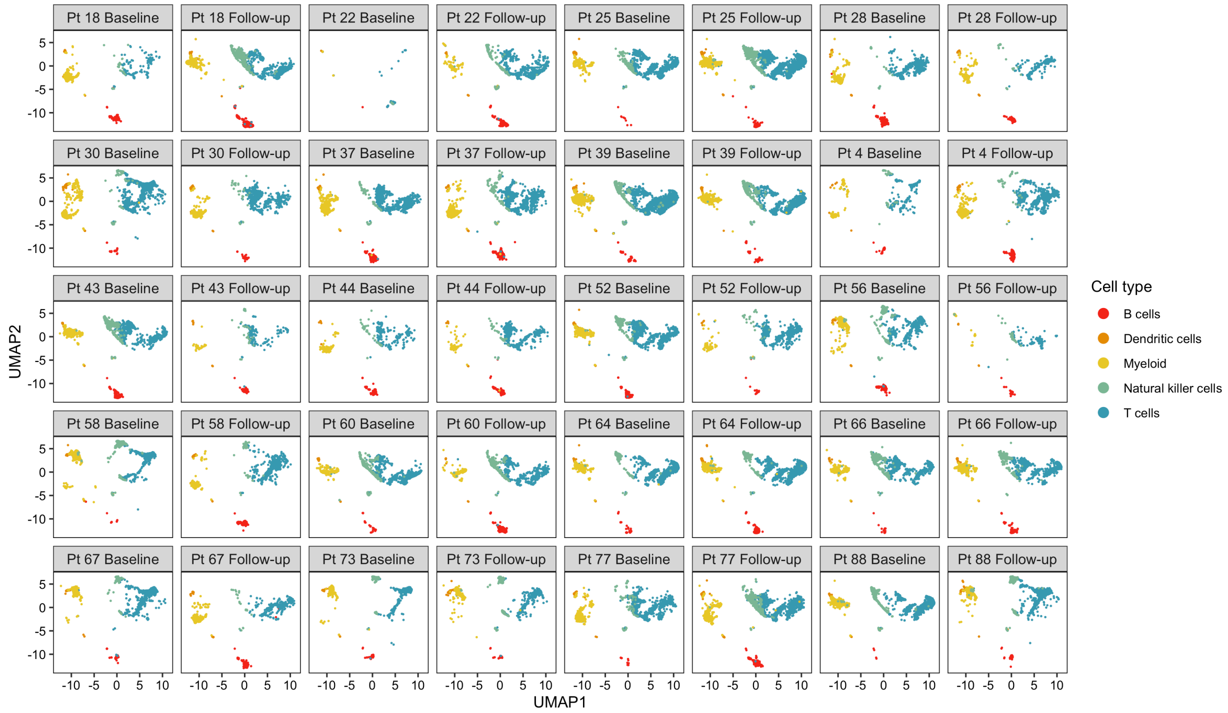

Supplementary Figure 1. Individualized uniform manifold approximation and projection (UMAP) of each sample studied showing the overall number of cells analyzed and the relative abundance of each cell subsets.

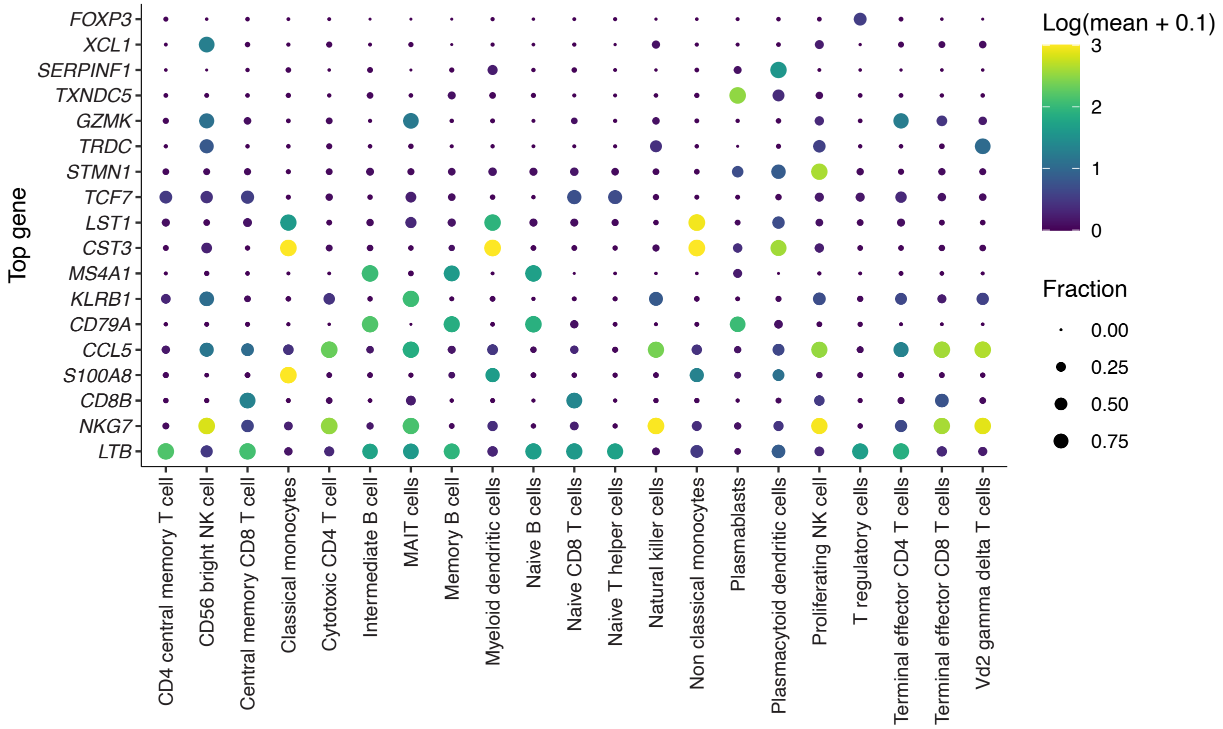

Supplementary Figure 2. Top genes most specifically expressed by each cell type. Dot plot showing the mean gene expression and percentage of expressed cells in each cell type. Genes expressed by less than 10% of cells were excluded. Using the top_markers function from the monocle3 R package, the gene with the highest pseudo R-squared value, a measure of how well the gene expression model fits the categorical data relative to the null model, are shown for each cell type.

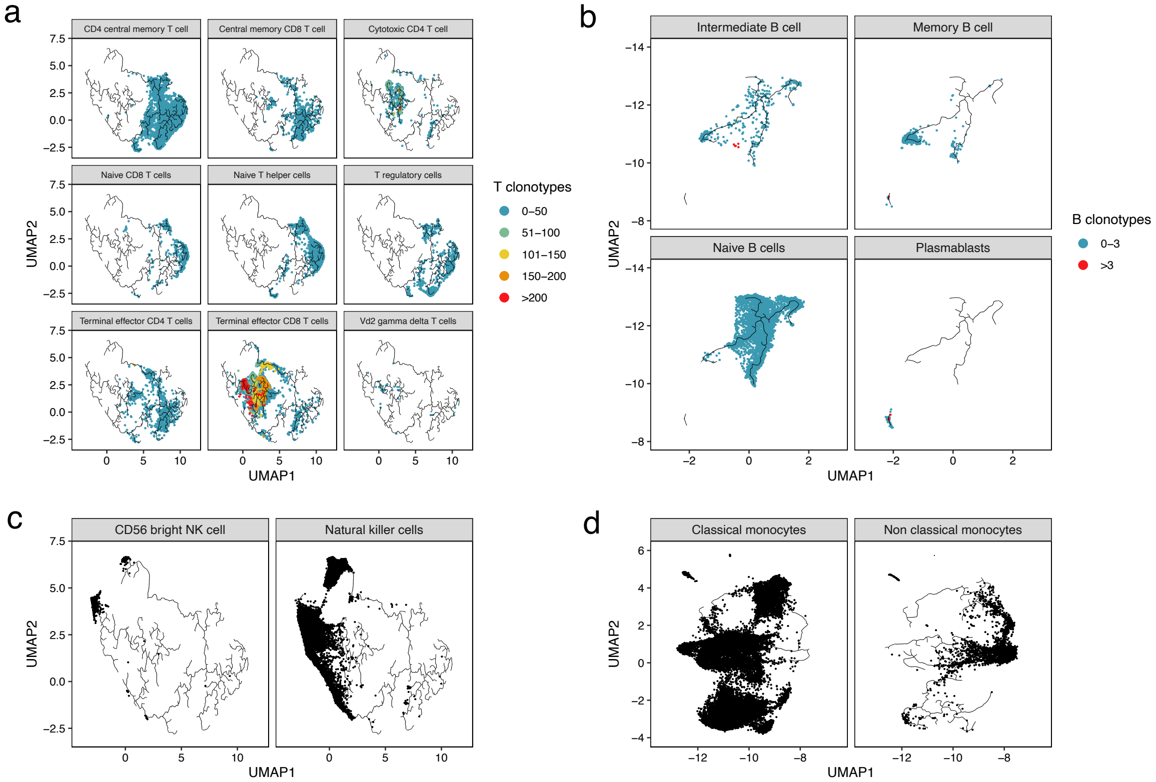

Supplementary Figure 3. Aggregate uniform manifold approximation and projection (UMAP) and trajectory analysis for all 24 samples. a) UMAP of T cells and b) B cells color-coded by VDJ clonality, where expanded indicates that cells T or B cell receptor was presented in more than one cell. Single-cell trajectories are represented by black lines. c) UMAP of NK and d) myeloid cells.

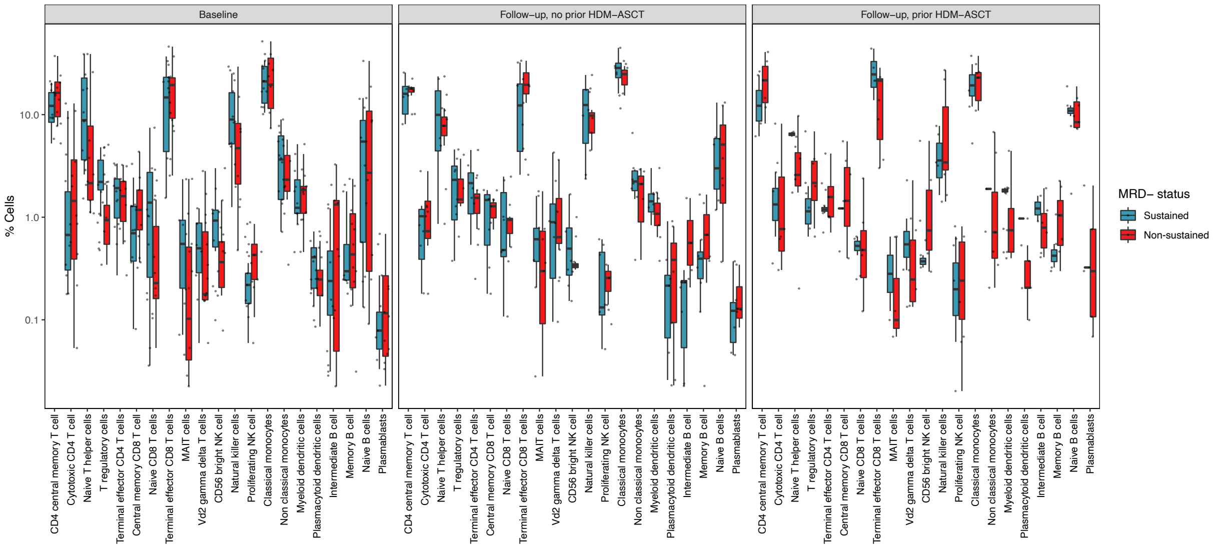

Supplementary Figure 4. Boxplots comparing the distribution of cell frequency by single-cell RNA sequencing (scRNAseq) across all cell types for baseline and follow-up samples aggregated by prior history of high-dose melphalan autologous stem cell transplant (HDM-ASCT) and minimal residual disease (MRD) negativity. N = 7 sustained MRD- (5 no prior HDM ASCT, 2 prior HDM ASCT) and 7 non-sustained MRD- (4 no prior HDM ASCT, 3 prior HDM ASCT) patient samples. In the box plot, the ends of the whiskers represent 1.5 times the interquartile range, the center represents the median, and the bounds of the box represent the first and third quartiles.

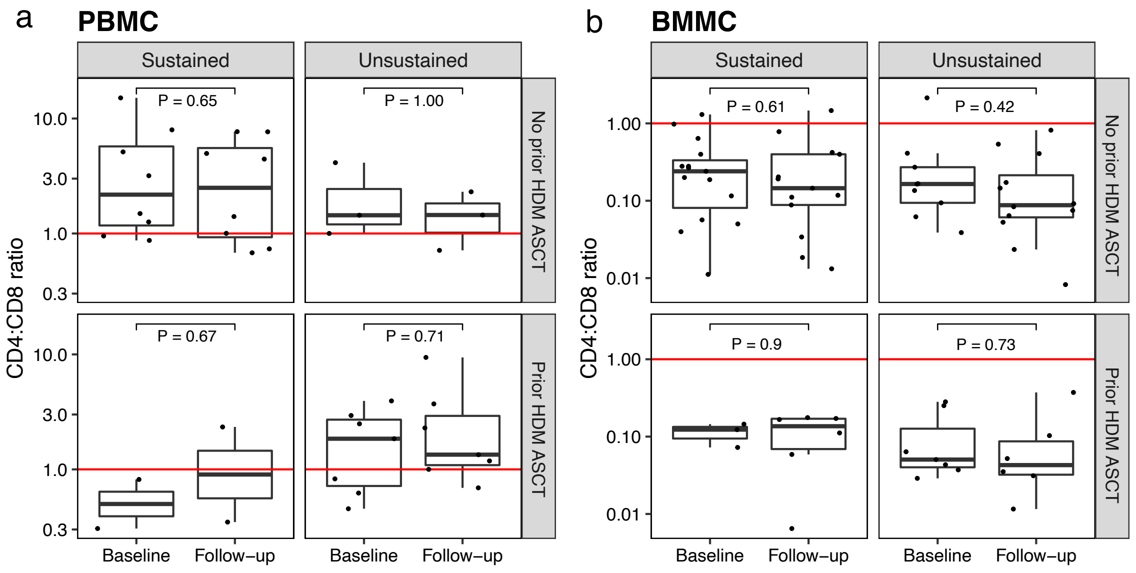

Supplementary Figure 5. A higher ratio of CD4/CD8 lymphocytes is observed in patients with sustained minimal residual disease (MRD) negativity and no prior history of transplant. Box plot showing the distribution of CD4/CD8 ratios in a) the peripheral blood mononuclear cells (PBMC) measured by single-cell RNA sequencing (scRNAseq) and b) the bone marrow mononuclear cells (BMMC) measured by CyTOF and grouped by time point, MRD negative status, and history of HDM-ASCT. The red line indicates a ratio equal to one. Two-sided Wilcoxon Rank Sum test P values are shown. For PBMC scRNAseq analysis, n = 7 sustained MRD- (5 no prior HDM ASCT, 2 prior HDM ASCT) and 7 non-sustained MRD- (4 no prior HDM ASCT, 3 prior HDM ASCT) patient samples. For BMMC CyTOF analysis, n = 7 sustained MRD- (5 no prior HDM ASCT, 2 prior HDM ASCT) and 7 non-sustained MRD- (4 no prior HDM ASCT, 3 prior HDM ASCT) patient samples. In the box plot, the ends of the whiskers represent 1.5 times the interquartile range, the center represents the median, and the bounds of the box represent the first and third quartiles. HDM ASCT, high-dose melphalan autologous stem cell transplant.

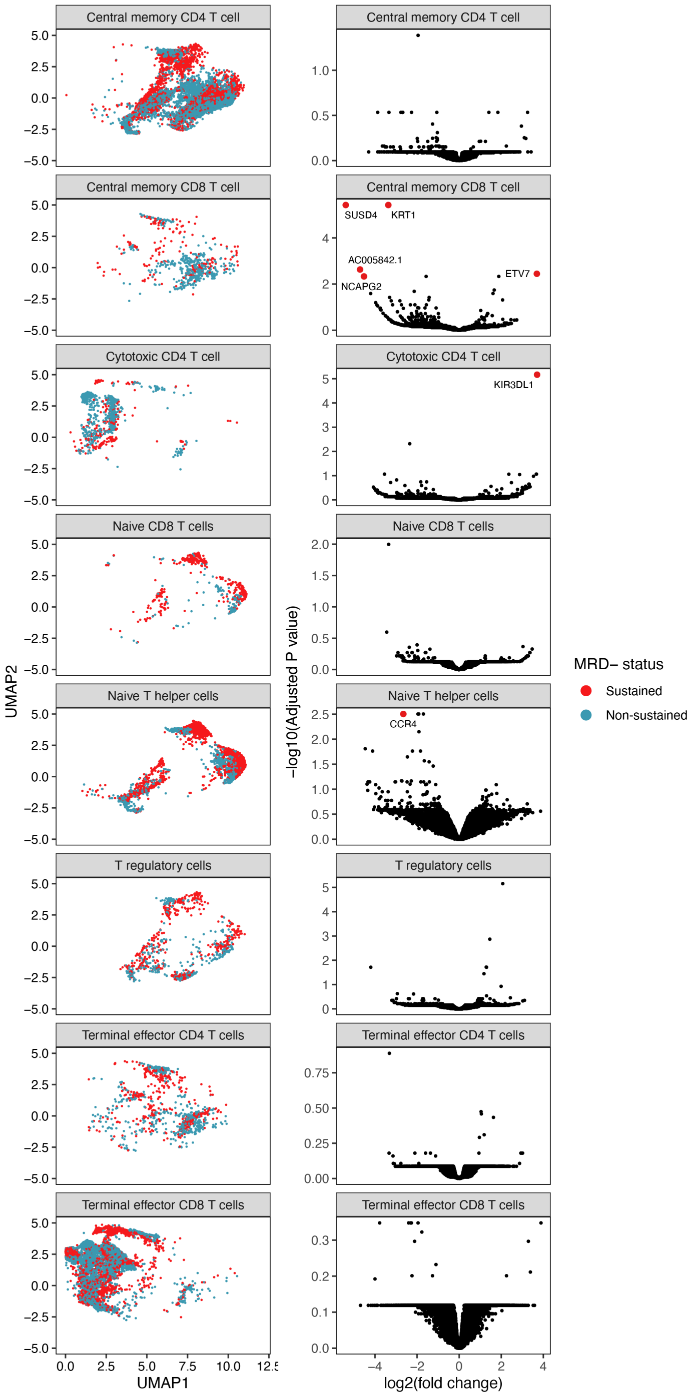

Supplementary Figure 6. uniform manifold approximation and projection (UMAP) of baseline T cell subclusters and volcano plots of differential gene expressed genes between patients achieving sustained versus non-sustained minimal residual disease (MRD) negativity before maintenance therapy. In the volcano plots, red points correspond to differentially abundant genes with Benjamini Hochberg adjusted P values < 0.01 and log fold change > 2.5 or < -2.5 using the EdgeR-LRT pseudobulk method from the Libra R package. A negative log fold change indicates increased expression in non-sustained MRD negativity while a positive log fold change indicates increased expression in sustained MRD negativity.

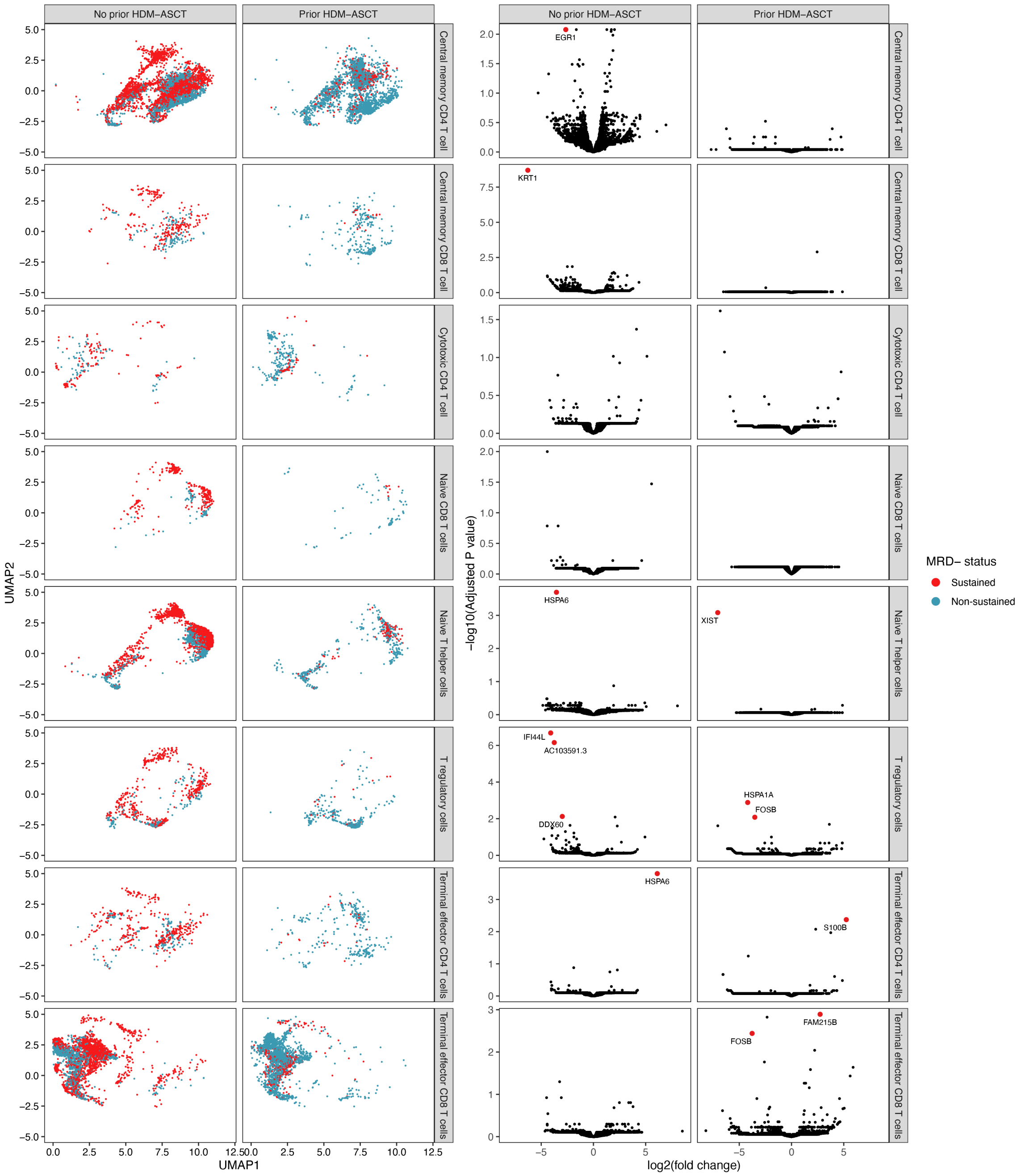

Supplementary Figure 7. uniform manifold approximation and projection (UMAP) of follow-up T cell subclusters and volcano plots of differentially expressed genes between patients achieving sustained versus non-sustained minimal residual disease (MRD) negativity 1 year after maintenance therapy. In the volcano plots, red points correspond to differentially abundant genes with Benjamini Hochberg adjusted P values < 0.01 and log fold change > 2.5 or < -2.5 using the EdgeR-LRT pseudobulk method from the Libra R package. A negative log fold change indicates increased expression in non-sustained MRD negativity while a positive log fold change indicates increased expression in sustained MRD negativity. HDM ASCT, high-dose melphalan autologous stem cell transplant.

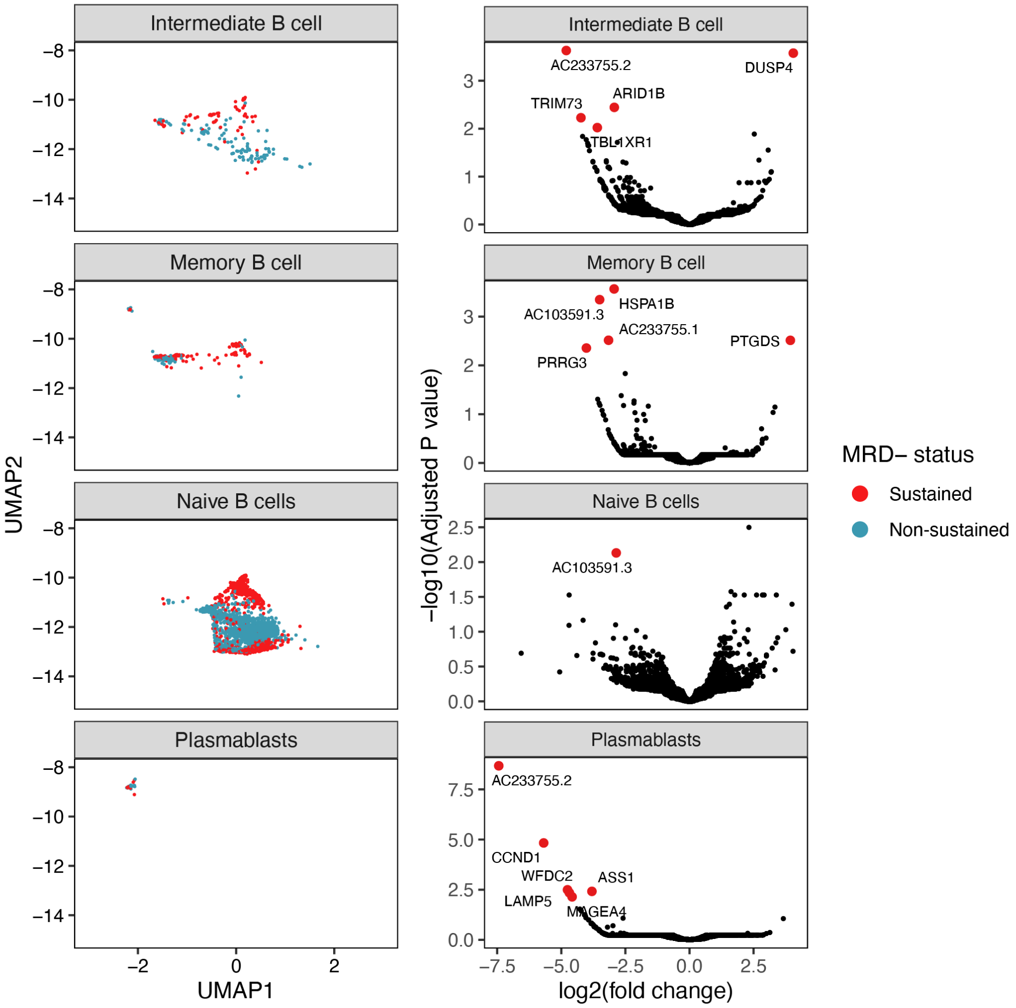

Supplementary Figure 8. uniform manifold approximation and projection (UMAP) of baseline B cell subclusters and volcano plots of differentially expressed genes between patients achieving sustained versus non-sustained minimal residual disease (MRD) negativity before maintenance therapy. In the volcano plots, red points correspond to differentially abundant genes with Benjamini Hochberg adjusted P values < 0.01 and log fold change > 2.5 or < -2.5 using the EdgeR-LRT pseudobulk method from the Libra R package. A negative log fold change indicates increased expression in non-sustained MRD negativity while a positive log fold change indicates increased expression in sustained MRD negativity.

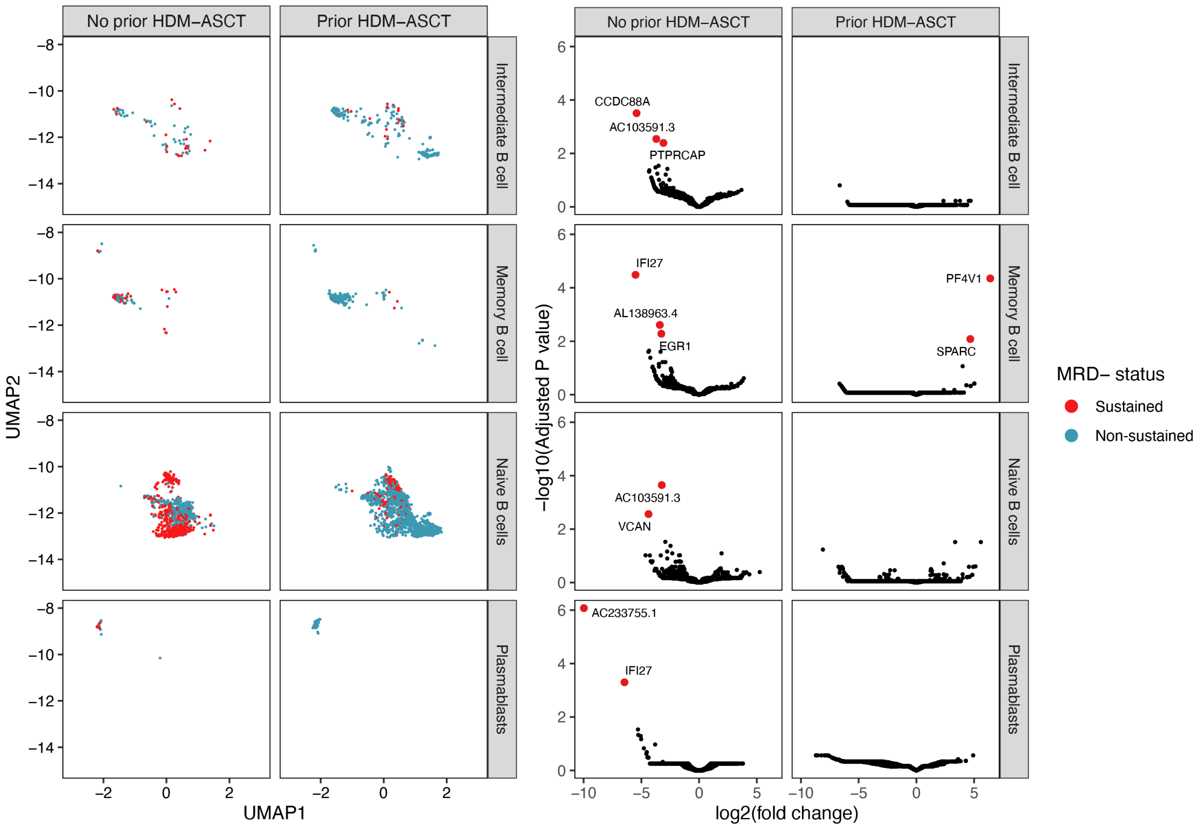

Supplementary Figure 9. uniform manifold approximation and projection (UMAP) of follow-up B cell subclusters and volcano plots of differentially expressed genes between patients achieving sustained versus non-sustained minimal residual disease (MRD) negativity 1 year after maintenance therapy. In the volcano plots, red points correspond to differentially abundant genes with Benjamini Hochberg adjusted P values < 0.01 and log fold change > 2.5 or < -2.5 using the EdgeR-LRT pseudobulk method from the Libra R package. A negative log fold change indicates increased expression in non-sustained MRD negativity while a positive log fold change indicates increased expression in sustained MRD negativity. HDM ASCT, high-dose melphalan autologous stem cell transplant.

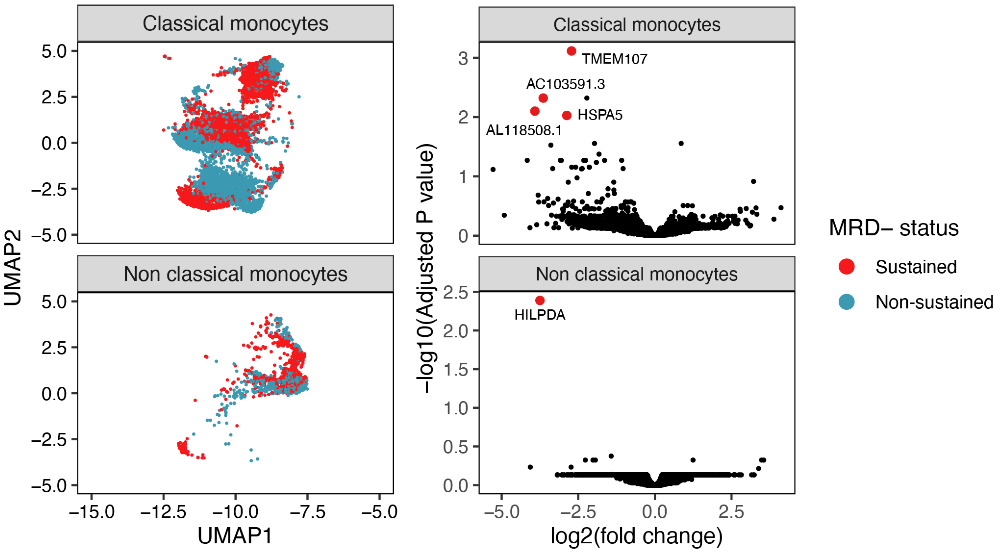

Supplementary Figure 10. uniform manifold approximation and projection (UMAP) of myeloid cell subclusters and volcano plots of differentially expressed genes between patients achieving sustained versus non-sustained minimal residual disease (MRD) negativity before maintenance therapy. In the volcano plots, red points correspond to differentially abundant genes with Benjamini Hochberg adjusted P values < 0.01 and log fold change > 2.5 or < -2.5 using the EdgeR-LRT pseudobulk method from the Libra R package. A negative log fold change indicates increased expression in non-sustained MRD negativity while a positive log fold change indicates increased expression in sustained MRD negativity.

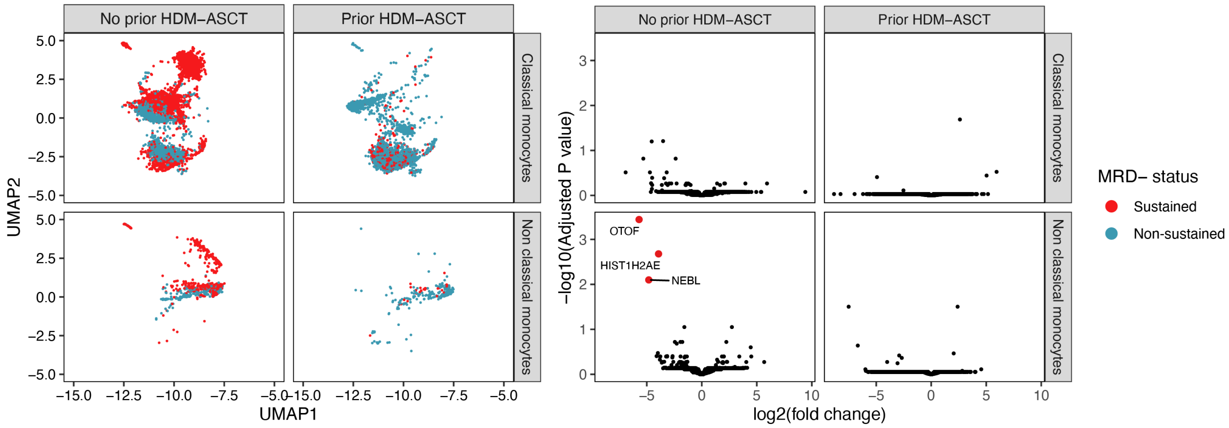

Supplementary Figure 11. uniform manifold approximation and projection (UMAP) of follow-up myeloid cell subclusters and volcano plots of differentially expressed genes between patients achieving sustained versus non-sustained minimal residual disease (MRD) negativity 1 year after maintenance therapy. In the volcano plots, red points correspond to differentially abundant genes with Benjamini Hochberg adjusted P values < 0.01 and log fold change > 2.5 or < -2.5 using the EdgeR-LRT pseudobulk method from the Libra R package. A negative log fold change indicates increased expression in non-sustained MRD negativity while a positive log fold change indicates increased expression in sustained MRD negativity.

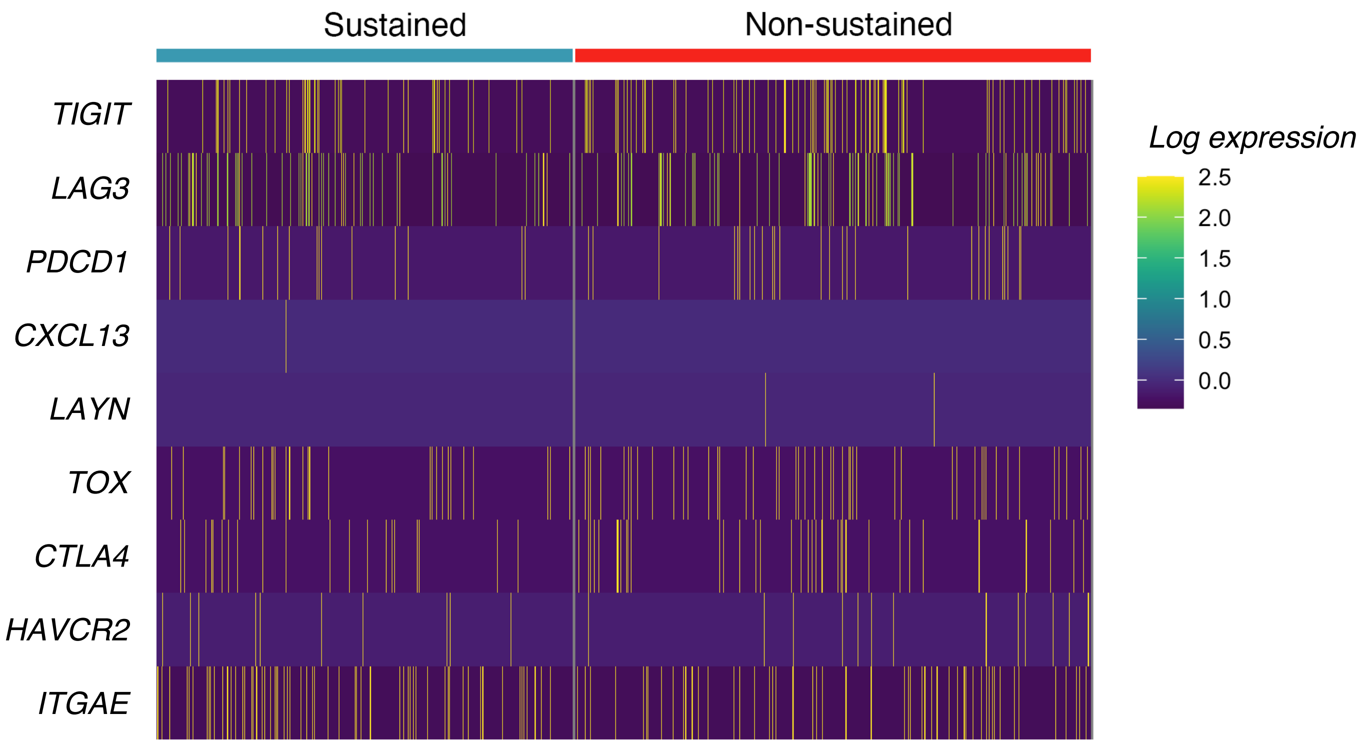

Supplementary Figure 12. Heatmap of normalized, single-cell expression of genes associated with exhaustion among T cells grouped by minimal residual disease (MRD) negative status.

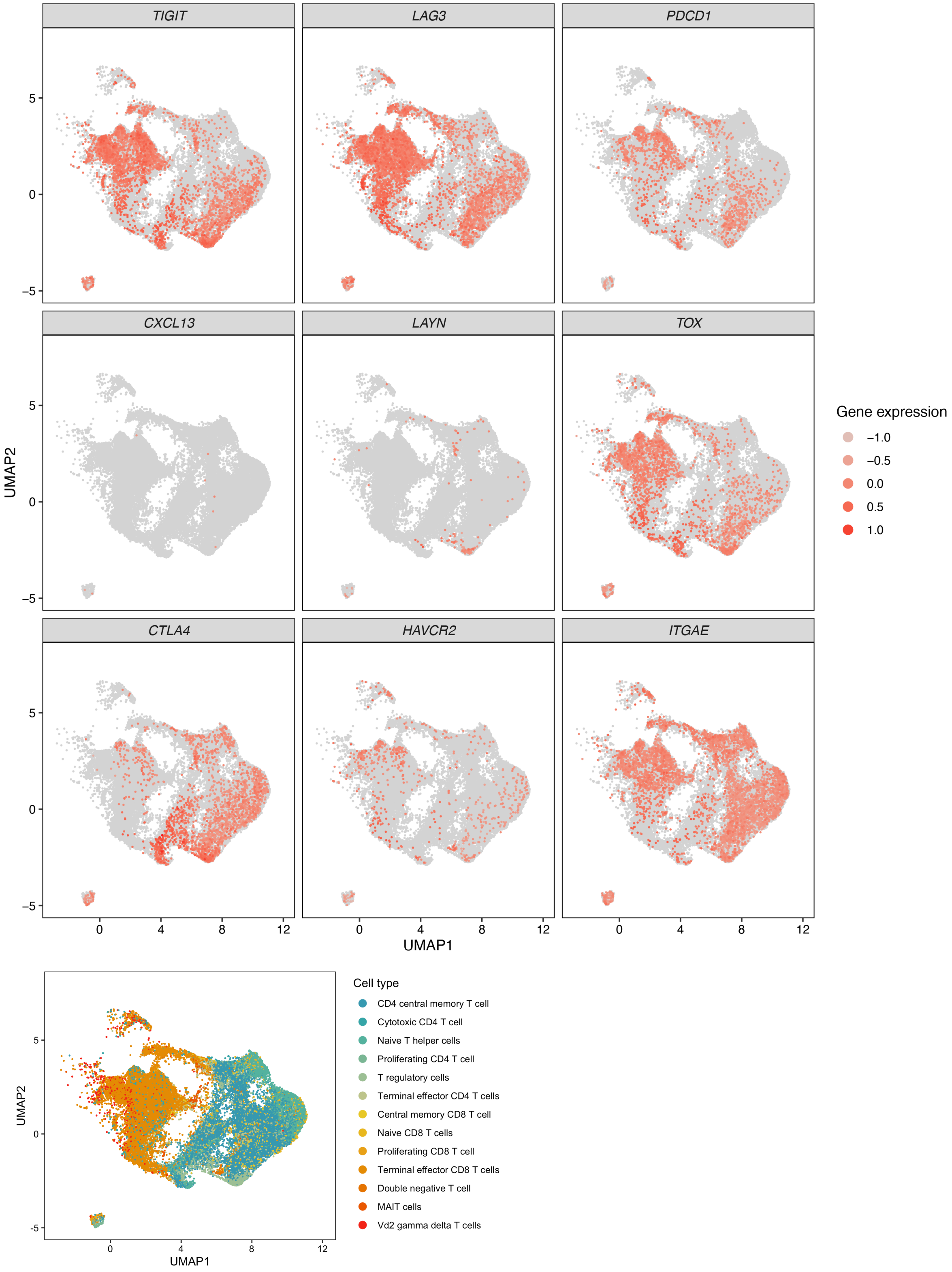

Supplementary Figure 13. uniform manifold approximation and projection (UMAP) of normalized single-cell expression of genes associated with exhaustion among T cells. UMAP colored by cell type is provided for reference.

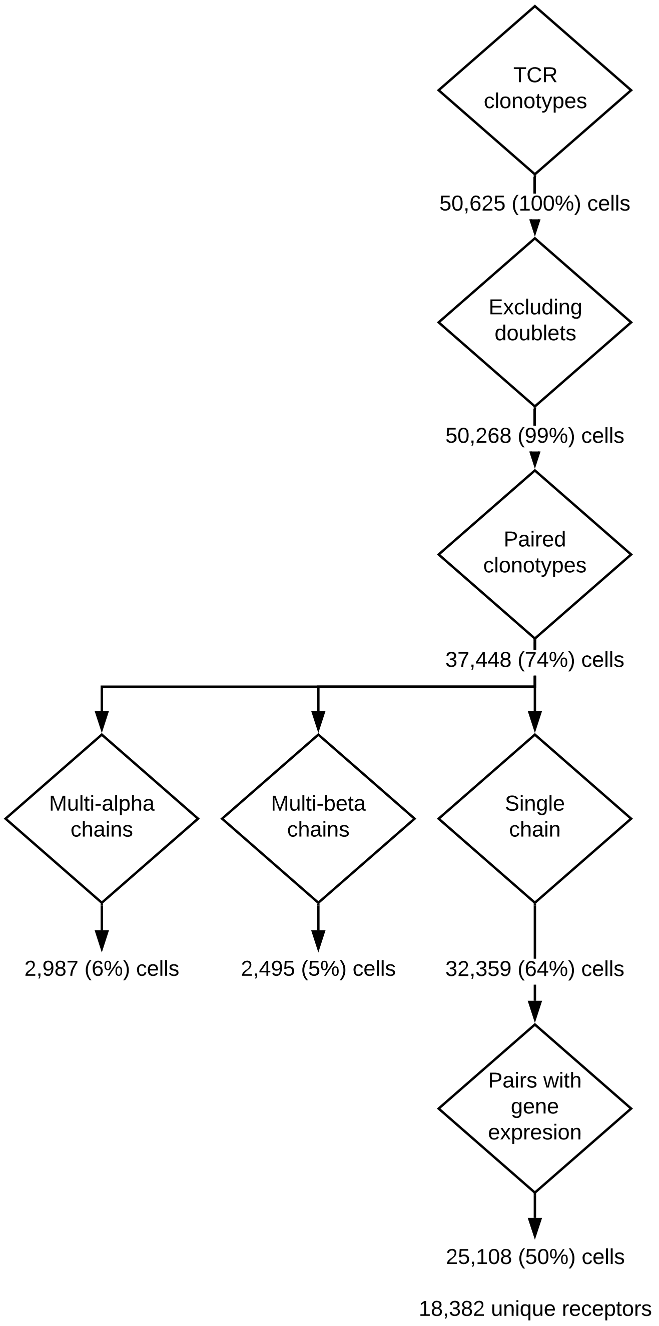

Supplementary Figure 14. Number of cells excluded from single-cell T cell receptor (TCR) analysis after quality filters were applied. A doublet was defined as a single-cell barcode associated with both a T and B cell receptor chain. Paired clonotype was defined as a single-cell barcode associated with an alpha and beta T cell receptor. Only TCR clonotypes with a single alpha and beta chain that paired with gene expression were considered in the final analysis.

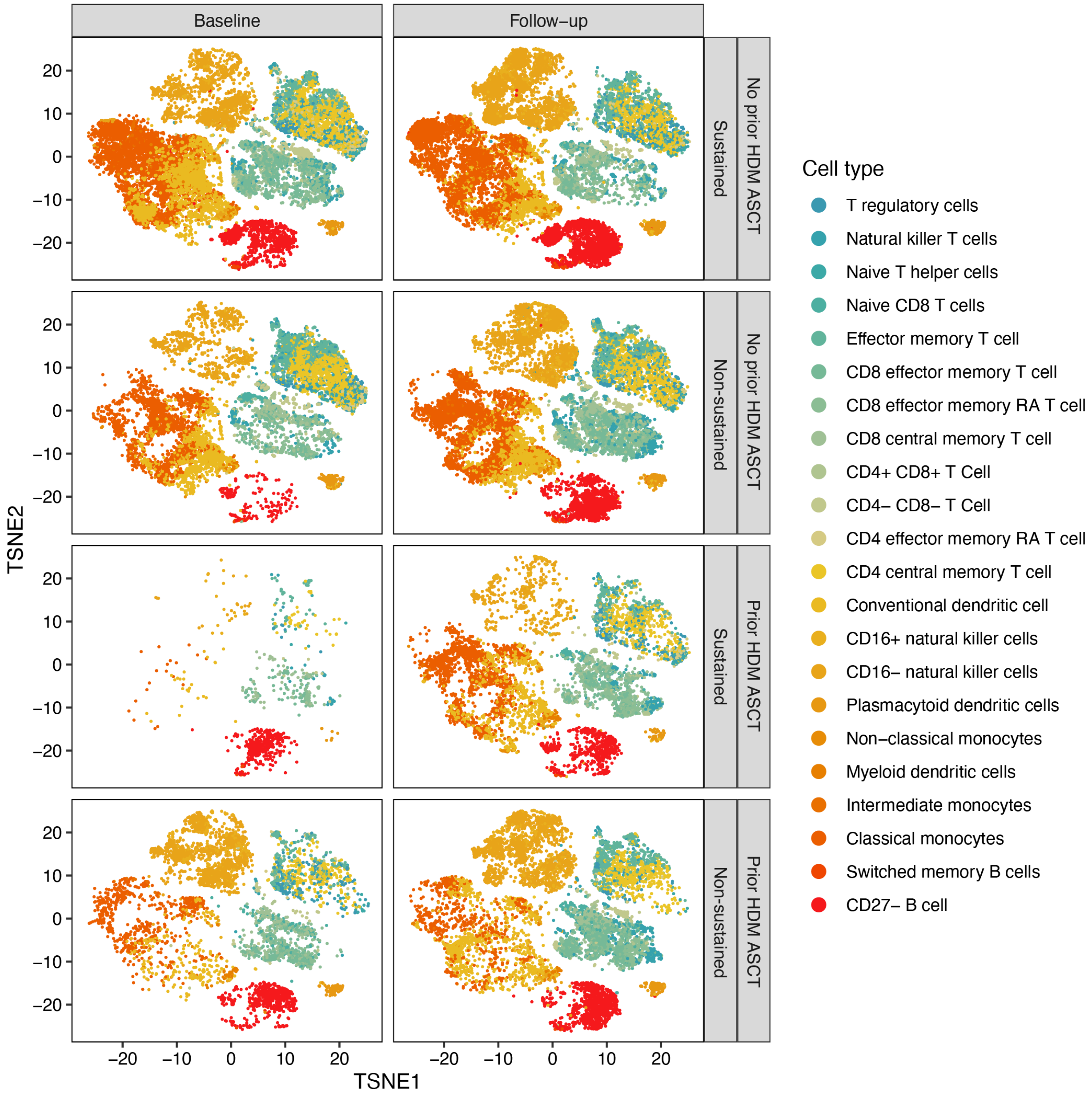

Supplementary Figure 15. tSNE plots of CyTOF data according to time point, transplant history and minimal residual disease (MRD) negative status. HDM ASCT, high-dose melphalan autologous stem cell transplant.

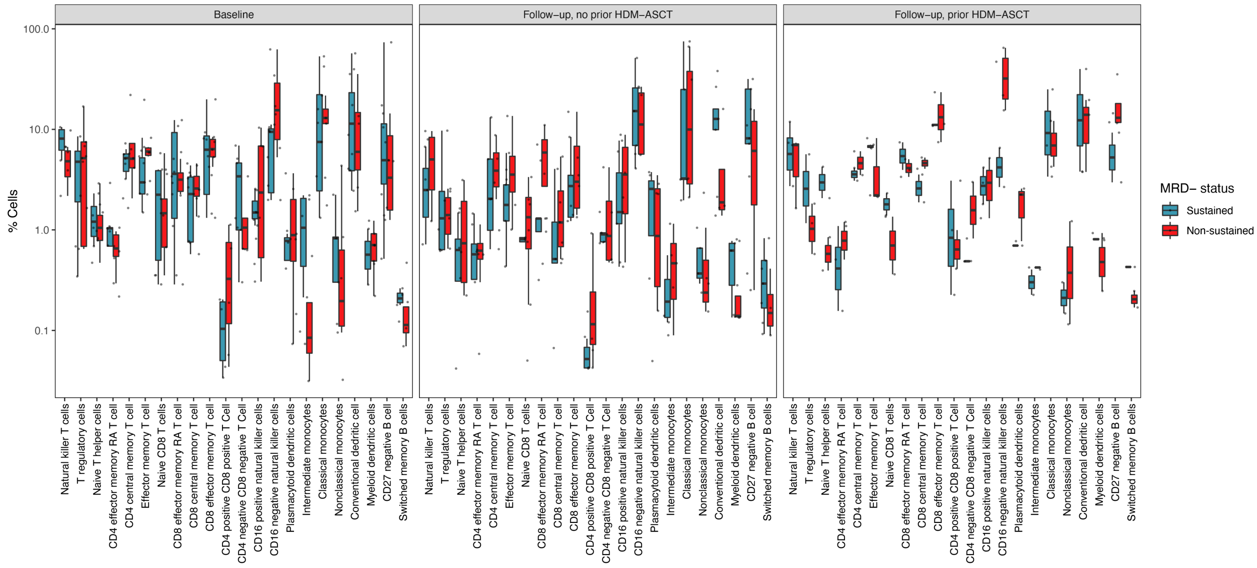

Supplementary Figure 16. Boxplots comparing the distribution of cell frequency by CyTOF across all cell types for baseline and follow-up samples aggregated by prior history of transplant and minimal residual disease (MRD) negative status. N = 7 sustained MRD- (5 no prior HDM ASCT, 2 prior HDM ASCT) and 7 non-sustained MRD- (4 no prior HDM ASCT, 3 prior HDM ASCT) patient samples. In the box plot, the ends of the whiskers represent 1.5 times the interquartile range, the center represents the median, and the bounds of the box represent the first and third quartiles. HDM ASCT, high-dose melphalan autologous stem cell transplant.

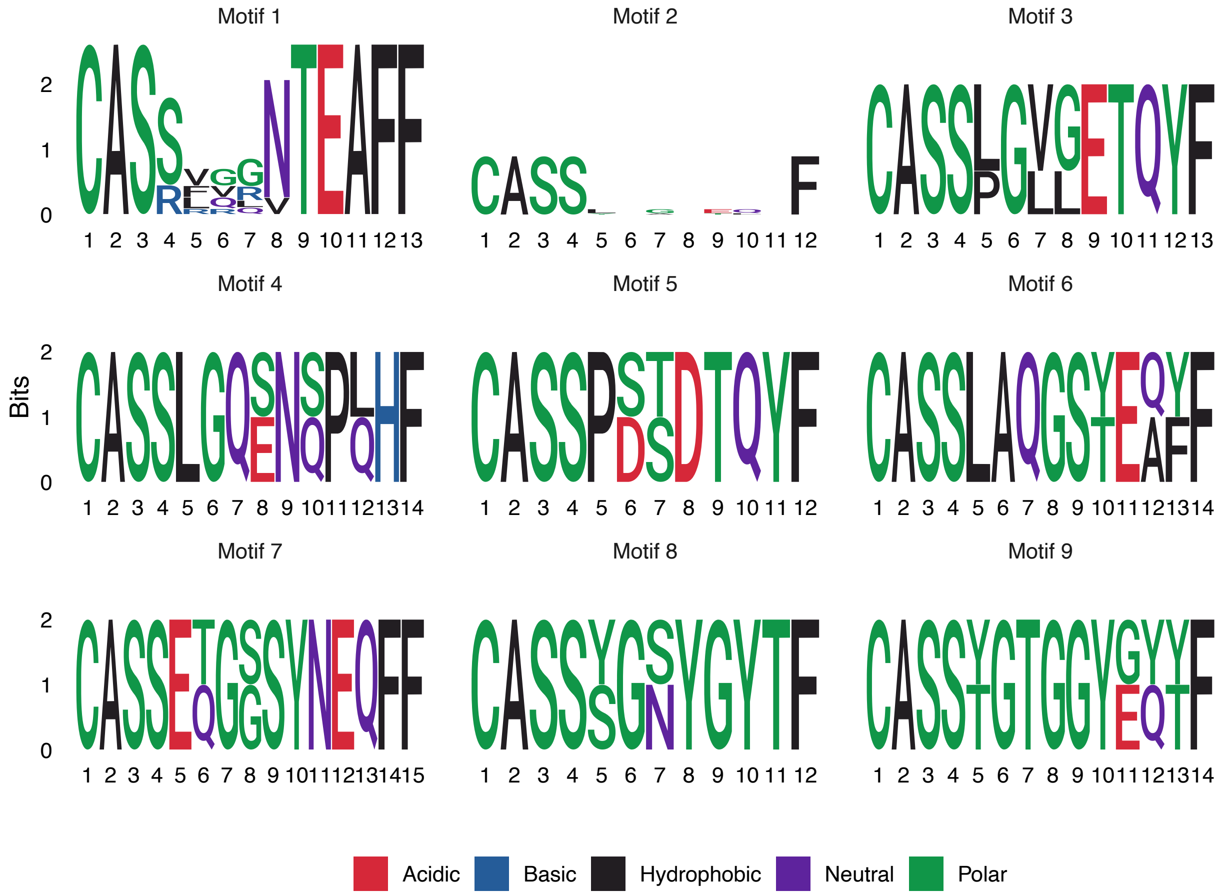

Supplementary Figure 17. Sequence logos of amino acid sequence motifs identified among the differentially abundant T cell receptor β Complementarity-Determining Region 3 sequences between sustained and non-sustained minimal residual disease (MRD) negative samples. Refer to Source Data for additional details regarding the original sequences.

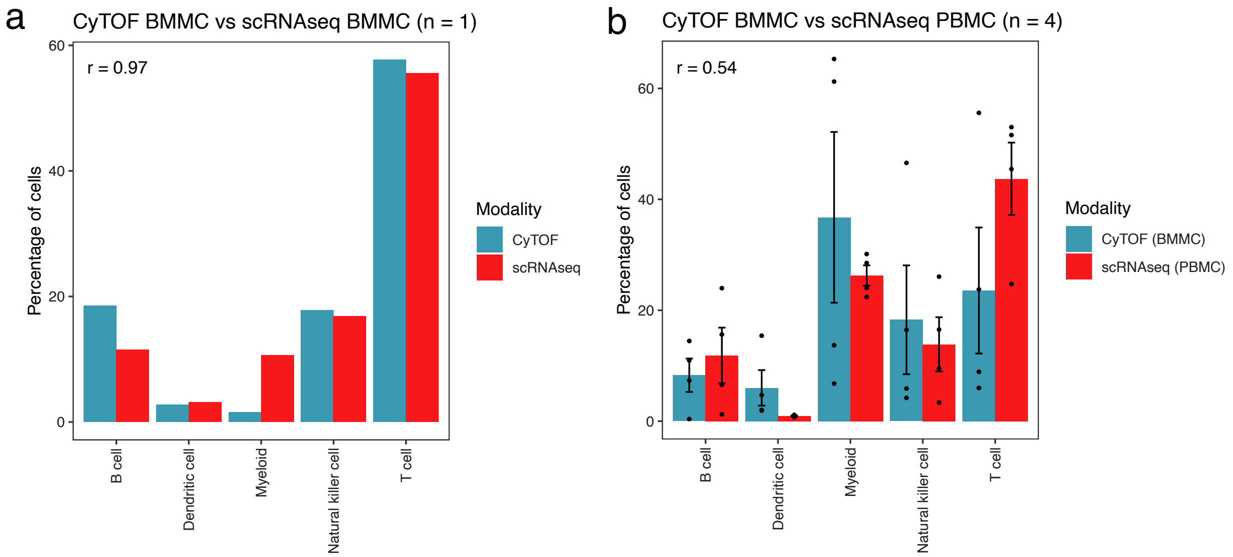

Supplementary Figure 18. Comparison between CyTOF and single-cell RNA sequencing (scRNAseq). a) Bar plot comparing the percentage of BMMCs measured by CyTOF and scRNAseq from the same sample. b) Bar plot comparing the mean percentage of BMMCs measured by CyTOF and percentage of PBMCs measured by scRNAseq collected on the same day from 4 separate individuals. Error bars represent standard error. Pearson's correlation (r) is shown in the plots. BMMC, bone marrow mononuclear cells; PBMC, Peripheral blood mononuclear cells

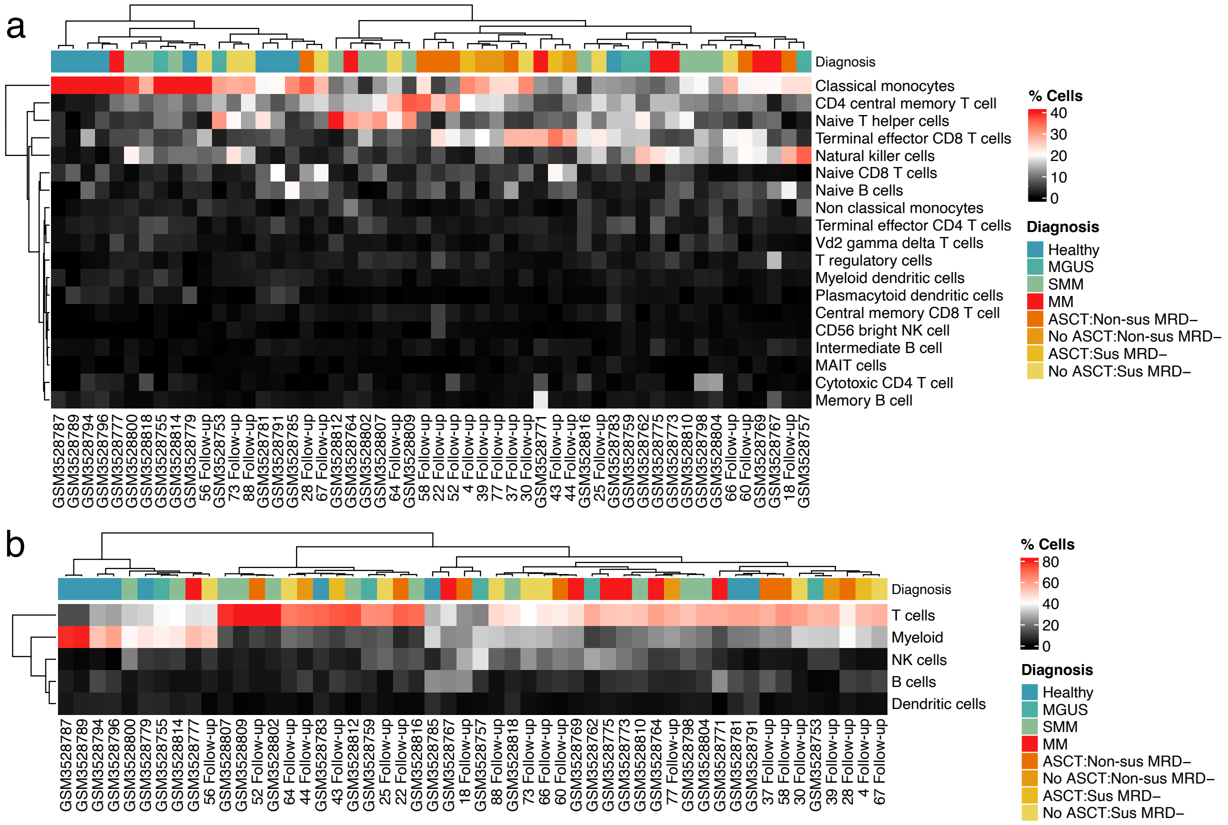

Supplementary Figure 19. Comparison of immune cell frequency in the bone marrow and blood among healthy donors as well as patients with various stages of myeloma measured by single-cell RNA sequencing (scRNAseq). Heatmap showing the mean cell frequency by a) cell type and b) cell group in healthy bone marrow, precursor myeloma, untreated myeloma as well as blood from patients after receiving lenalidomide maintenance. Hierarchical clustering was performed using Ward's minimum variance method. N = 9 healthy donors, 5 patients with MGUS, 11 patients with SMM, and 7 patients with untreated MM, 20 patients with sustained MRD- (16 no prior HDM ASCT, 4 prior HDM ASCT), and 20 patients with non-sustained MRD- (6 no prior HDM ASCT, 14 prior HDM ASCT). Non-sus, Non-sustained MRD negative; Sus, Sustained MRD negative; ASCT, autologous stem cell transplant.

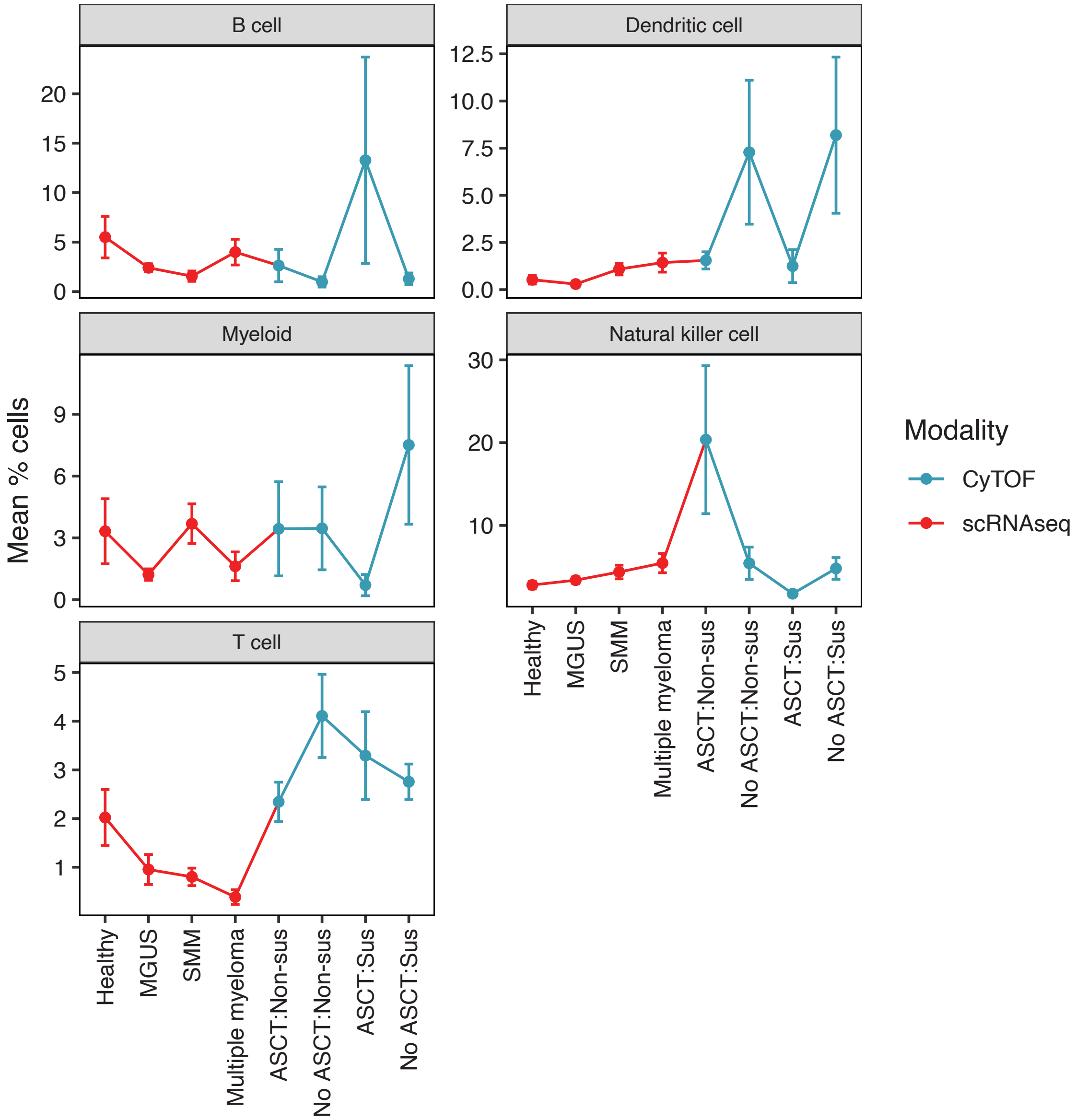

Supplementary Figure 20. Change in immune cell frequency in the bone marrow among healthy donors as well as patients with various stages of myeloma measured by single-cell RNA sequencing (scRNAseq) and CyTOF. Line plot showing the mean and standard error of the cell frequency of cell types in healthy bone marrow and untreated myeloma as well as blood from patients after receiving lenalidomide maintenance. N = 9 healthy donors, 5 patients with MGUS, 11 patients with SMM, and 7 patients with untreated MM, 7 patients with sustained minimal residual disease negativity (MRD-) (5 no prior HDM ASCT, 2 prior HDM ASCT) and 7 patients with non-sustained minimal residual disease negativity (MRD-) (4 no prior HDM ASCT, 3 prior HDM ASCT). Non-sus, Non-sustained MRD negative; Sus, Sustained MRD negative; ASCT, autologous stem cell transplant.

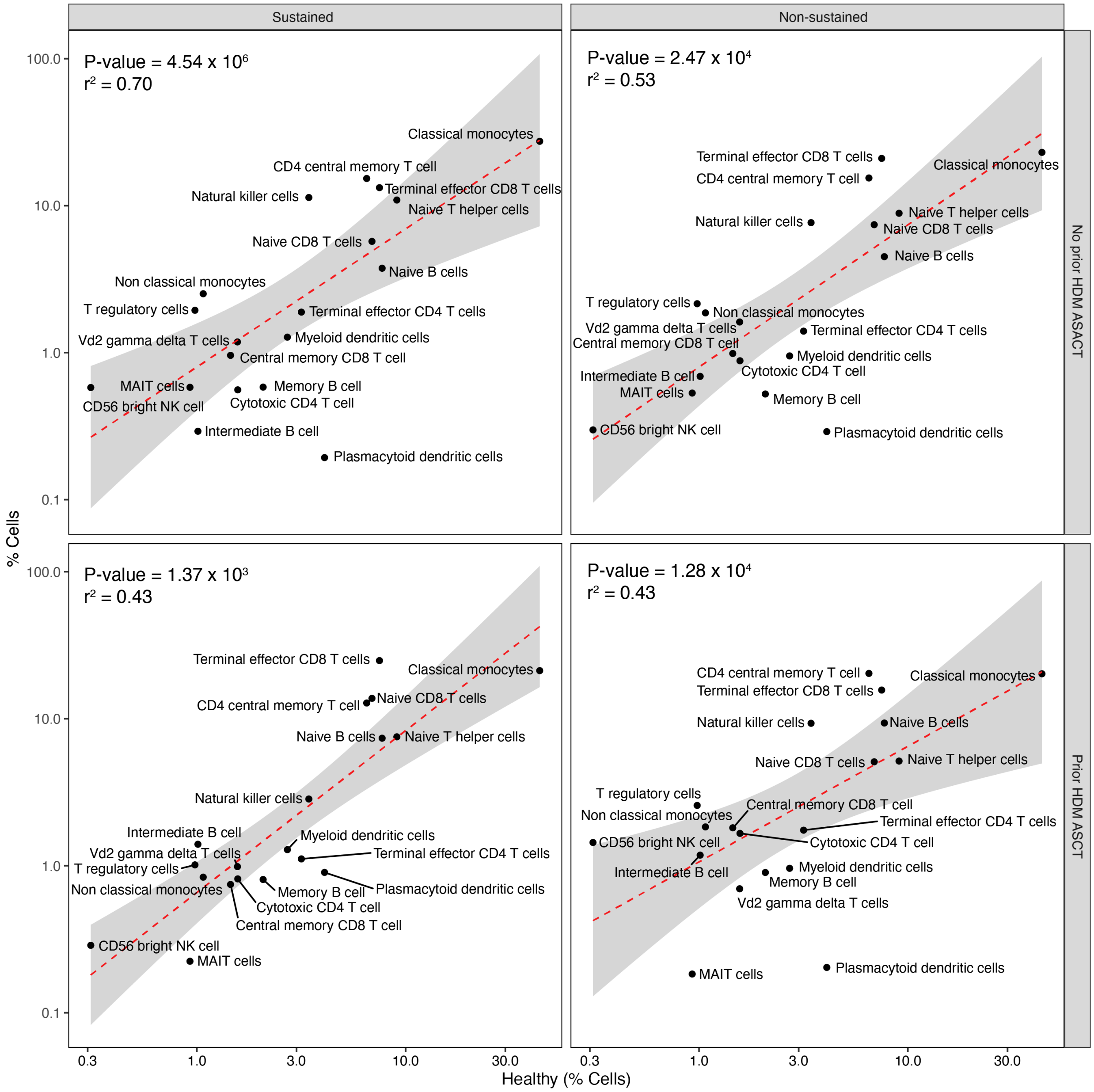

Supplementary Figure 21. Scatter plots and linear regression analysis showing correlation between immune cell frequency in healthy bone marrow versus myeloma blood with and without prior high-dose melphalan autologous stem cell transplant and achieving or not achieving sustained minimal residual disease (MRD) negativity. Line of best fit is shown in red and grey represents 95% confidence interval.
