# Supplementary material for: Immunophenotypic correlates of sustained MRD negativity in patients with multiple myeloma
Source: Nat Commun. 2023 Sep 2;14:5335. doi: 10.1038/s41467-023-40966-8 (PMC10475030; doi:10.1038/s41467-023-40966-8)
Supplement: Supplementary file 3 — Description of Additional Supplementary Files [file 41467_2023_40966_MOESM3_ESM.pdf]

Description of Additional Supplementary Files

Supplementary Data 1. Characteristics of individual study patients (n = 23).

Supplementary Data 2. CD4/CD8 ratios in peripheral blood mononuclear cells (PBMC) measured by single-cell RNA sequencing (scRNAseq) and bone marrow mononuclear cells (BMMC) measured by CyTOF

Supplementary Data 3. Percentage of terminal effector memory T cells (TEM) expressing a known cancer exhaustion marker by single-cell RNA sequencing.

Supplementary Data 4. Filtered VDJ clonotypes per cell from single-cell RNA sequencing.

Supplementary Data 5. Single-cell T cell receptor Gini index of CD4+ and CD8+ T cells. A higher Gini index corresponds to greater receptor diversity.

Supplementary Data 6. Differentially abundant circulating T cell receptor β CDR3 amino acid sequences specific to patients achieving either sustained or unsustained MRD negativity. Antigen, known antigen specificity reported in VDJdb, McPAS-TCR, PIRD TBAdb, or LymphoSeqDB; Phenotype, T cell phenoptype in scRNAseq dataset; Number of patients, number of patients with detected CDR3β amino acid sequence in this study; GLIPH motif, amino acid sequence motif revealed by GLIPH indicating increased likelihood of shared antigen specifity (see Figure S17); Prevelance, the frequency (%) of the CDR3β amino acid among 55 healthy individuals ages 0-90 years from LymphoSeqDB; Generation probability, the probability of generating the CDR3β amino acid computed by OLGA.

Supplementary Data 7. Single-cell RNA sequencing cell counts automatically classified by Azimuth

Supplementary Data 8. Single-cell RNA sequencing cell counts of external dataset (Gene Expression Omnibus ID GSE124310) automatically classified by Azimuth.

Supplementary Data 9. CyTOF labeling stategy implemented by Astrolabe software.The table should be interpreted as a hierarchy. For example, CCR7+ CD4 central memory T cells are a subset of CD4+ T cells (CD45RA-), which are a subset of CD4+ T cells, which are a subset of CD3+ CD19- CD56- CD33- T cells.

Supplementary Data 10. CyTOF cell counts automatically classified by Astrolabe

Supplementary Data 11. TCRβ sequencing sample metrics.
