# Supplementary material for: Immunophenotypic correlates of sustained MRD negativity in patients with multiple myeloma
Source: Nat Commun. 2023 Sep 2;14:5335. doi: 10.1038/s41467-023-40966-8 (PMC10475030; doi:10.1038/s41467-023-40966-8)
Supplement: Supplementary file 5 — Reporting Summary [file 41467_2023_40966_MOESM5_ESM.pdf]

nature portfolio

Corresponding author(s): David G. Coffey and C. Ola Landgren

Last updated by author(s): 2023-6-29

Reporting Summary

Nature Portfolio wishes to improve the reproducibility of the work that we publish. This form provides structure for consistency and transparency in reporting. For further information on Nature Portfolio policies, see our Editorial Policies and the Editorial Policy Checklist.

Statistics

For all statistical analyses, confirm that the following items are present in the figure legend, table legend, main text, or Methods section.

n/a	Confirmed	
☐	☒	The exact sample size (*n*) for each experimental group/condition, given as a discrete number and unit of measurement
☐	☒	A statement on whether measurements were taken from distinct samples or whether the same sample was measured repeatedly
☐	☒	The statistical test(s) used AND whether they are one- or two-sided<br>*Only common tests should be described solely by name; describe more complex techniques in the Methods section.*
☐	☒	A description of all covariates tested
☐	☒	A description of any assumptions or corrections, such as tests of normality and adjustment for multiple comparisons
☐	☒	A full description of the statistical parameters including central tendency (e.g. means) or other basic estimates (e.g. regression coefficient) AND variation (e.g. standard deviation) or associated estimates of uncertainty (e.g. confidence intervals)
☐	☒	For null hypothesis testing, the test statistic (e.g. *F*, *t*, *r*) with confidence intervals, effect sizes, degrees of freedom and *P* value noted<br>*Give P values as exact values whenever suitable.*
☒	☐	For Bayesian analysis, information on the choice of priors and Markov chain Monte Carlo settings
☒	☐	For hierarchical and complex designs, identification of the appropriate level for tests and full reporting of outcomes
☐	☒	Estimates of effect sizes (e.g. Cohen's *d*, Pearson's *r*), indicating how they were calculated

Our web collection on statistics for biologists contains articles on many of the points above.

Software and code

Policy information about availability of computer code

Data collection	Synapse (Sage Bionetworks) was used for data collection.
Data analysis	All analyses were performed in R v4.1. Single-cell RNA sequencing analyses (scRNAseq) were conducted using cell ranger v4.0.0 (10X Genomics), monocle v3.0, Seurat v4.0, and Libra v1.0. TCRβ sequencing analyses were performed using GLIPH v1, LymphoSeq v1.14.1, immunarch v0.6.9, and TCellPack v2.2 (https://github.com/davidcoffey/TCellPack). CyTOF analyses were performed using the Astrolabe Mass Cytometry Platform (Astrolabe Diagnostics, Inc.), FlowSOM v2.8 and EdgeR v3.40.2. Custom R scripts are available on GitHub (https://github.com/UM-Myeloma-Genomics/Immunophenotypic-correlates-of-sustained-MRD-negativity).

For manuscripts utilizing custom algorithms or software that are central to the research but not yet described in published literature, software must be made available to editors and reviewers. We strongly encourage code deposition in a community repository (e.g. GitHub). See the Nature Portfolio guidelines for submitting code & software for further information.

Data

Policy information about availability of data

All manuscripts must include a data availability statement. This statement should provide the following information, where applicable:

- Accession codes, unique identifiers, or web links for publicly available datasets
- A description of any restrictions on data availability
- For clinical datasets or third party data, please ensure that the statement adheres to our policy

Processed scRNAseq has been made publicly available through CReSCENT: CanceR Single Cell ExpressioN Toolkit (https://crescent.cloud, CRES-P31), a web portal for

standardized analysis and exploration of scRNAseq data from cancer studies. The raw scRNAseq data are protected and are not available due to data privacy laws. scRNAseq and CyTOF cell counts are available in the Source Data provided with this paper. Single-cell V(D)J calls are available in the Source Data file. TCR β sequencing is available within the ImmuneAccess database (https://clients.adaptivebiotech.com/immuneaccess, DOI 10.21417/DGC2023NC). Previously published scRNAseq from the bone marrow of healthy donors and patients MGUS, SMM, and MM were accessed from the Gene Expression Omnibus under accession code GSE124310. Queried T cell receptor databases are accessible through the R packages LymphoSeqDB (https://bioconductor.org/packages/LymphoSeqDB) and immunarch (https://immunarch.com/).

Research involving human participants, their data, or biological material

Policy information about studies with human participants or human data. See also policy information about sex, gender (identity/presentation), and sexual orientation and race, ethnicity and racism.

Reporting on sex and gender	Both self-reported males and females were included in this study. Males were more frequent, likely because multiple myeloma more commonly affects males. Sex and gender were not considered in the study design, nor did we conduct specific analyses comparing the two due to lack of statistical power.
Reporting on race, ethnicity, or other socially relevant groupings	We did not collect information about race, ethnicity or socially relevant groupings for this study.
Population characteristics	Relevant covariates are reported in supplementary data. These include age, sex, cancer stage, prior treatment, and treatment response.
Recruitment	Refer to the previous publication reporting on the clinical outcomes of this study (Diamond, B. et al. Dynamics of minimal residual disease in patients with multiple myeloma on continuous lenalidomide maintenance: a single-arm, single-centre, phase 2 trial. Lancet Haematol 8, e422–e432 (2021).
Ethics oversight	Memorial Sloan Kettering Cancer Center Institutional Review Board

Note that full information on the approval of the study protocol must also be provided in the manuscript.

Field-specific reporting

Please select the one below that is the best fit for your research. If you are not sure, read the appropriate sections before making your selection.

[x] Life sciences [ ] Behavioural & social sciences [ ] Ecological, evolutionary & environmental sciences

For a reference copy of the document with all sections, see nature.com/documents/nr-reporting-summary-flat.pdf

Life sciences study design

All studies must disclose on these points even when the disclosure is negative.

Sample size	Immune profiling was performed on 23 patients with newly diagnosed multiple myeloma who participated in the single-arm, phase II clinical trial evaluating the effect of lenalidomide as maintenance therapy. This sample size was felt to be sufficient given that a prior study of the same number of patients (5 with MGUS, 11 with SMM, and 7 with untreated MM) showed significant differences within the immune microenvironment by scRNAseq and CyTOF during disease transformation (Zavidij, O. et al. Single-cell RNA sequencing reveals compromised immune microenvironment in precursor stages of multiple myeloma. Nat Cancer 1–14 (2020) doi:10.1038/s43018-020-0053-3).
Data exclusions	For the single-cell RNA sequencing analysis, cells with less than 500 unique molecular identifiers, less than 250 aligned genes, or greater than 20% mitochondrial genes detected were excluded. For the CyTOF analysis, cell types were excluded if there were less than 3 cells in at least half of all samples.
Replication	CyTOF and single-cell RNAseq were directly compared on the same bone marrow sample from a single patient to determine the reproducibility of both assays. Additionally, CyTOF on bone marrow and single-cell RNAseq on peripheral blood collected from the same patient were also directly compared to determine quantitative differences of immune cell subsets within the different body compartments for four patients. The results of these comparisons are reported in supplemental figure 18. For the remaining samples, sufficient material was available to successfully analyze each sample by CyTOF, scRNAseq, and TCRβ as a single replicate.
Randomization	Since we investigated samples collected from an observational study, randomization was not relevant to its design.
Blinding	When selecting biospecimen for this study, investigators were not blinded to the patient's minimal residual disease status, but were blinded to all other clinical history (choice of induction therapy, history of transplant, cytogenetics, and cancer stage)

Reporting for specific materials, systems and methods

We require information from authors about some types of materials, experimental systems and methods used in many studies. Here, indicate whether each material, system or method listed is relevant to your study. If you are not sure if a list item applies to your research, read the appropriate section before selecting a response.

Materials & experimental systems

n/a	Involved in the study
[ ]	[x] Antibodies
[x]	[ ] Eukaryotic cell lines
[x]	[ ] Palaeontology and archaeology
[x]	[ ] Animals and other organisms
[x]	[ ] Clinical data
[x]	[ ] Dual use research of concern
[x]	[ ] Plants

Methods

n/a	Involved in the study
[x]	[ ] ChIP-seq
[x]	[ ] Flow cytometry
[x]	[ ] MRI-based neuroimaging

Antibodies

Antibodies used	The manufacturer, catalog number, and clone identifier of all monoclonal antibodies used for CyTOF are shown in Supplementary Table 1.
Validation	All CyTOF antibodies were validated by the manufacturer. Specifically, testing was performed on multiple cell and tissue types with a variety of known expression levels using multiple applications as a cross-check. Comparison to existing antibody clones was also performed.
